# Miscellaneous

**Published:** 1898-05

**Authors:** 


### MISCELLANEOUS.

### LIQUID AIR.

One of the latest achivements of physics to excite wide-spread interest is the liquefaction of air on an extensive scale. Air had been liquefied as early as 1880 by Dewar, of the Royal Institution, and, indeed, as far back as 1777, Pictet, of Geneva, succeeded in liquefying oxygen. In both cases the liquefaction was accomplished by the use of an auxiliary liquefied gas, the evaporation of which produced a degree of cold sufficient to liquefy the air or gas. In 1890 Mr. Tripler, of New York, discovered a process not alone superior to that previously employed, but also less expensive. He secured the liquefaction of air entirely by its own expansion, after subjecting it to a pressure of 2,000 pounds. The sudden expansion of the compressed air produces such a degree of cold that a part of the air—about one-third—is rendered liquid. Liquid air at atmospheric pressure has a temperature of 191° C. (about 312° F.); it is a slightly opalescent liquid, and can be poured from one vessel to another just as any other liquid substance.

Professor Barker, of the University of Pennsylvania, who has been chiefly instrumental in bringing Mr. Tripler's discovery before the scientific public, has made an extensive series of experiments with the liquid air, which, by the new process, is obtainable in large quantities. Some of these experiments border almost on the magical, and fascinated his hearers.

Exposed to the atmosphere, liquid air boils and steams like boiling water. The degree of cold engendered is so intense that almost everything brought in contact with the liquid air is instantly frozen. Mercury becomes solid; soft rubber-tubing is rendered so brittle that it breaks into fragments; a piece of beefsteak placed in it becomes stony hard and can be broken with a hammer like glass. Upon pure metals, such as gold, silver and copper, liquid air has no effect. It supports combustion, though it itself, like ordinary air, is not inflammable. On account of the unequal boiling points of liquid oxygen and liquid nitrogen, that of the latter being much higher, the nitrogen is given off more rapidly, and the liquid becomes progressively richer in oxygen. The slight opalescence of the liquid is due to frozen water and carbon dioxide.

It need hardly be said that the intense cold is destructive to the skin and produces painful blisters. Professor Barker told the writer that he had a burn from a hot plate on one thumb and a blister from liquid air on the other, and remarked that he could scarcely tell the difference.

Regarding the possible uses of liquid air, it seems that Mr. Tripler hopes to employ it as a motive power and for purposes of refrigeration. The latter can, it is said, be accomplished at less expense than by the ordinary freezing process.

To science, liquid air offers a profitable field for experimentation.

Most substances, when exposed to a low temperature, change their nature more or less; and being able to produce by means of liquid air a temperature as low nearly as 200° C., science will have a valuable opportunity for studies in this direction. It has been shown that the electric resistance of metals is diminished by cold; if the resistance can be entirely nullified, it will be possible to transmit powerful currents of electricity to great distances without loss. And it has, indeed, been suggested to surround conductors with an envelope of liquid air to reduce the resistance to a minimum.

As far as our own science is concerned, Professor Barker gave utterance to the hope that liquid air might find in it many uses. As a germicide and preservative it would be far more efficient than freezing—as Professor Barker quaintly remarked in conversation, "a quart of liquid air could be left at the house every morning and placed in the refrigerator." An additional advantage in this respect is that it gives off no moisture, but yields up oxygen, which purifies the air. In the sick-room it could be employed as a cooling agent, as well as for purifying the atmosphere. Whether it will be useful as a local anesthetic is questionable, as the degree of cold is too great; yet a means of regulating this may be found. For freezing tissue for histologic purposes it probably possesses no superiority over compressed carbon dioxid.—*Philadelphia Polyclinic.*

---

### THE POSSIBILITIES OF ANTITOXIN IN DIPHTHERIA.

There is no difference of opinion now among medical men regarding the efficiency of diphtheria antitoxin. Statistics have abundantly proved the decreased death-rate resulting from its use, although there may be found an occasional doubter who refuses to believe any kind of evidence regarding this new remedy.

Having had something to do with the introduction of antitoxin for the use of the Contagious Department of Harper Hospital of this city, and having watched its administration from the beginning, the writer believes that a very much better percentage of result is attained with the early use of antitoxin in situations where hygienic conditions prevail, such as are found in a well-conducted hospital or even in the average private practice.

Antitoxin was first used in Harper Hospital in the fall and winter of 1894 in a sporadic tentative way. Forty-four cases of diphtheria were treated with the Aronson and Behring's serum, with four deaths (exclusive of three moribund on admission), and a death-rate accordingly of 9.1 per cent., when it happened that the supply of these became exhausted. At this juncture Parke, Davis & Co.'s diphtheria antitoxin was employed and found to be fully equal to any of the serums which had been previously employed; indeed, this is putting it too mildly, for not only did fewer disagreeable sequelæ follow its hypodermic administration, but cases progressed manifestly better under it. Thus in the remaining period of 1895 there were 25 cases, all of whom were treated with this serum (with the exclusion of one moribund on admission). There was one death among these, death-rate accordingly being 4.1 per cent. Four were tracheotomy cases with four recoveries.

In 1896 there were 112 cases admitted to the diphtheria ward of the Contagious Hospital, 12 being subjected to intubation, 6 to tracheotomy, with only five deaths, "three of these being moribund cases when brought to the hospital, dying within a few hours of their arrival." There was, therefore, a mortality of 1.8 per cent.

For the present year, 1897, up to December, there have been treated 90 cases of diphtheria in the hospital, with 6 deaths. One case was certainly moribund on admission, another may be called doubtfully so, but without excluding the latter, the death-rate was 5.6 per cent. The antitoxin used was the same.

The notable diminution in the death-rate as above given led the Detroit Board of Health to decide to furnish diphtheria antitoxin gratuitously to persons too poor to pay for it, this also including patients under the charge of the city physicians, poor patients at Harper Hospital sent there by the Board of Health, at the Women's Hospital and at the Protestant Orphan Asylum. This was done from May 1, 1896, and the number of patients so treated up to February 28, 1897, close of the official year, was as follows:

| | Cases. | Deaths. | Mortality Rate. |
|---|---|---|---|
| With Antitoxin | 374 | 47 | 12.56 per cent. |
| Without Antitoxin | 467 | 163 | 34.90 per cent. |

In continuation of this series of observations are the following results from figures not yet made public, but kindly furnished me by the Board of Health. From March 1, 1897, up to December, the following cases came either under the notice or care of the Board:

| | Cases. | Deaths. | Mortality Rate. |
|---|---|---|---|
| With Antitoxin | 305 | 32 | 10.49 per cent. |
| Without Antitoxin | 632 | 192 | 30.39 per cent. |

The antitoxin employed by the Detroit Board of Health has been from the first that of Parke, Davis & Co.

These figures go to show that as experience in its use accumulates, and as both medical men and the public at large see the advantage arising from the use of antitoxin *early* in the diphtheritic invasion, there is attained a continued and steady improvement in results.

With patients in comfortable circumstances, assured of careful nursing, conscientious isolation, and the administration of a reliable antitoxin, there is very much to encourage the profession to expect a very close approach to the Harper Hospital figures—that is to say, a percentage of deaths not to exceed 6 or 7. This would certainly lift diphtheria out of the bad repute it has had hitherto of being one of our most fatal diseases.—DR. GEORGE SUTTIE, of Detroit, in *Louisville Med. Monthly*, Feb., 1898.

### SILKWORM GUT.

Dr. R. T. Morris, of New York, in the *Kansas City Medical Index*, says:

Silkworm gut is an instrument of the devil when used for buried permanent sutures. About three years ago I read an article on the use of silkworm gut by a surgeon whom I knew to be a careful observer, and I was tempted to give up my good-enough catgut for something better. In the course of six weeks I employed buried silkworm gut sutures in some twenty abdominal operations for patients who had put confidence in me and had entrusted their lives to my care. At the end of the six weeks I found that some of these buried silk knots were coming out in strange places, and some are coming out yet—three years elapsed. One patient died as the result of the burrowing of a silkworm gut knot in the pelvis several months after operation. Nearly all of the patients suffered in one way or another. The surgeon who wrote the paper advocating the use of the buried silkworm gut knot has written another paper saying that he is sorry. Meanwhile other surgeons are bringing discredit upon surgery by taking up this innocent looking suture material, which is so smooth and nice, but which refuses to become encapsulated in the tissues, and which comes out of the bladder or iliac vein just after the surgeon has written his paper. A recent advocate of the buried silkworm gut suture intimates that some of us did not use aseptic precaution, and that we did not choose the right sort of silkworm gut. To this I will reply that most of my wounds healed completely by primary union and the knots came out months or years afterward.

To those who say they have had no trouble with the buried silkworm gut knots, I wish to say that they have not taken the trouble to find out. The trouble is all on the part of the patients. The patient goes to one surgeon for operation, and to another one to complain. S› it requires two surgeons to write the history of buried silkworm guts.

---

### THE NEGRO PROBLEM.

Some one has written very entertainingly in an editorial in the *New York Medical Record* upon "The Physical Degeneracy of the Negro." He solves the "race problem," which has puzzled so many brains and been the cause of so much thought and words, in very short metre and very effectually. He claims that physical degeneracy will soon lead to the entire oblivion of the race. Those whom tuberculosis will not claim for its own will be carried away by venereal diseases, the latter having become so prevalent among them that it has rendered the female negro sterile to a large extent. And the greater the admixture of white blood, the smaller the negro's resistance to disease.

"The 'negro problem' is being slowly solved by busy little bacteria, working a slow but sure decay. Unless the negro becomes the ward of this great and good government, or is transported to some primitive country beyond the pale of civilization and its vices and allowed to recuperate, his doom is sure and certain. The negro problem will be solved by the forces of nature, and not by leather-lunged fanatics who shout themselves hoarse over effete theories of social equality.—*Colorado Med. Journal.*

### CANCER OF THE BREAST.

Dr. W. L. Rodman states that the results of Keen, Bull, Dennis, Weir, Halsted, and Powers, six American surgeons, who have within the year published their statistics in operations for cancer of the breast, show a mortality of less than one per cent. (656 operations and six deaths). He concludes his paper with the following propositions:

1. All mammary growths should be removed at once, for innocent tumors, carried for a long time, become a menace.
2. The complete operation should always be done in case of malignant disease.
3. In nearly every case it is simply impossible to detect enlarged glands until the axilla is opened. Keen says he cannot do so once in ten times.
4. The mortality should be, with average operators, about three per cent.
5. A radical operation should promise from 25 to 50 per cent. of permanent cures, according to the time when patients apply.
6. When in doubt operate; never wait for symptoms.—*Charlotte Medical Observer.*

### "KNOCK-OUT DROPS."

By this term is understood among the criminal classes the use of some potent drug which is introduced into a beverage without the victim's knowledge.

G. W. Henry (*Medical World,* February, 1898) investigated this subject while acting as coroner. He found that the drops were composed of hydrate of chloral and water, the high solubility of chloral making it peculiarly effective in this way, as a drachm of water will readily dissolve sixty grains of chloral. Consequently a two-drachm vial, which could be easily carried in the vest pocket, could be made to contain about 120 grains of chloral, and could be readily manipulated in the hand without attracting attention. He thinks it can be poured into a glass of whisky, wine, beer, or other drink, without noticeably affecting the taste or color. Chloral is also easily procured upon various pretexts, and is sold much more readily than some of the less potent poisons.—*Medicine.*

---

### GENERAL TONIC.

℞ Strychninæ sulphatis.................................... gr. 1-60.

Acidi phosphorici diluti.................................... m. v.

Ferri phosphatis.................................... gr. i.

Quininæ bisulphatis.................................... gr. i.

Glycerini.................................... ℥ ss.

Elix. aurantii, q. s.................................... ℥ ss.

M. et ft. solutio. Sig.—Take before each meal.

—*Winslow Anderson.*

---

Mercurial stomatitis is in many instances due to a dirty condition of the mouth, and this, frequently among those who ought to know better. If you will order your patient to clean his teeth every three hours, and oftener if he should eat anything between times, he will be far less liable to the pain and annoyance of stomatitis than if this simple rule is not observed. A soft tooth brush and a mildly alkaline lotion should be used.—*International Journal of Surgery.*

---

### DIURETIC PILL.

The following pill is highly recommended in cases of dropsy of cardiac origin:

℞ Scillæ, pulv.,
Digitalis, pulv.,
Caffeinæ citratis.................................... aa grs. xxx.
Hydrarg. chlor. mitis.................................... grs. v.

M. Ft. pil. no. xxx. Sig.—One pill three times daily, after meals.

*N. A. Practitioner.*

